# Supplementary material for: Power laws in pressure-induced structural change of glasses
Source: Nat Commun. 2020 Apr 24;11:2005. doi: 10.1038/s41467-020-15583-4 (PMC7181815; doi:10.1038/s41467-020-15583-4)
Supplement: Supplementary file 1 — Supplementary Information [file 41467_2020_15583_MOESM1_ESM.pdf]

Supplementary Information for

Power laws in pressure-induced structural change of glasses

Zhang, Qiao and Han

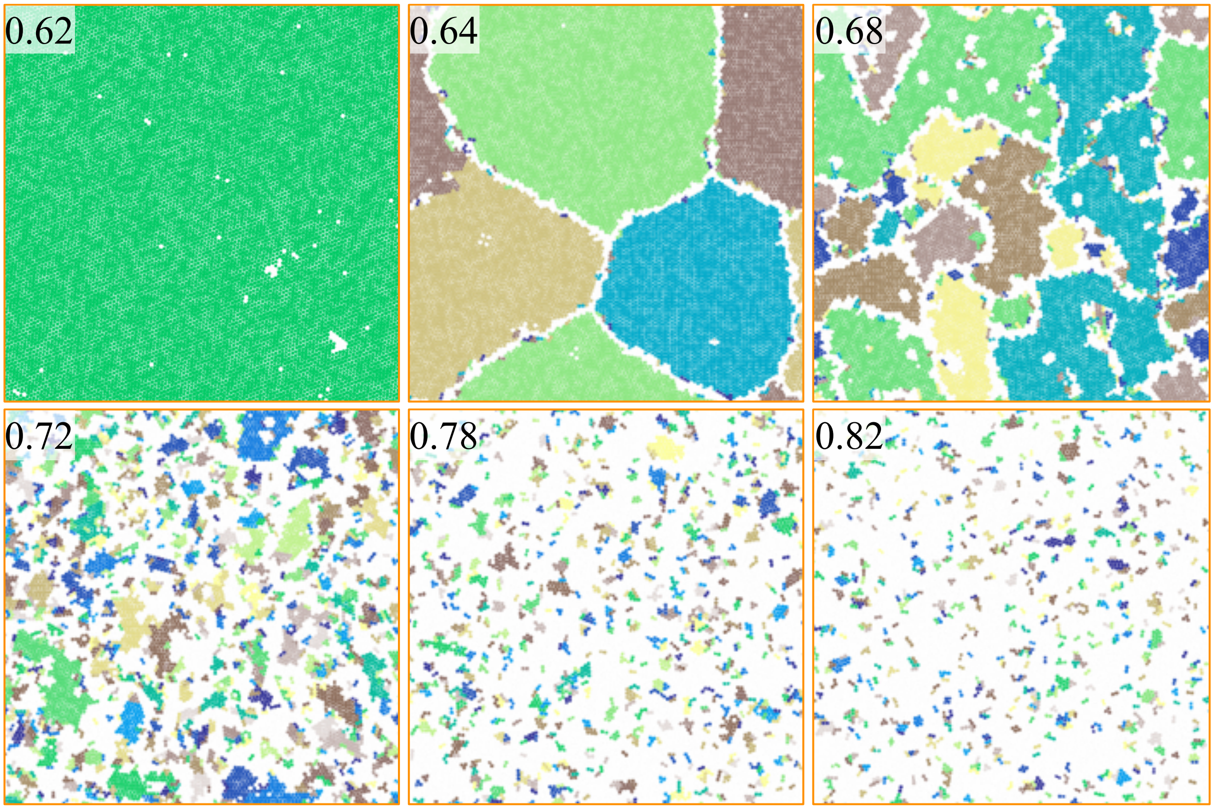

Supplementary Figure 1: **Representative snapshots of the compression-induced crystal-to-glass transition in the 2DSH system with** $(\eta, \lambda) = (0.5, 1.3)$. Structures at $\phi = 0.62$, 0.64, 0.68, 0.72, 0.78, and 0.82, respectively, corresponding to single crystal and five regimes in Fig. 2 [1]. Crystalline grains are labeled in colors, while disordered particles are labeled in white (see Method). Ref. [1] identified a sharp polycrystal-glass transition at $\phi = 0.70$, corresponding to a mean grain diameter of 17 particles.

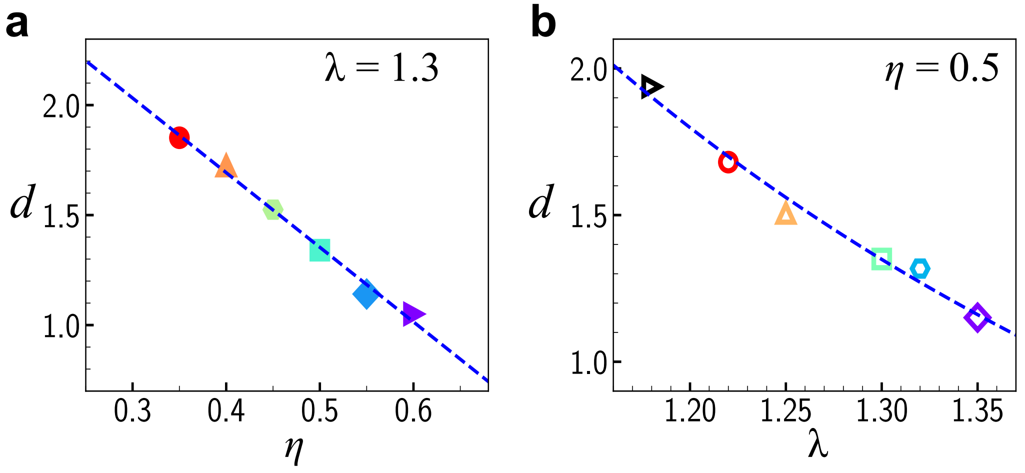

Supplementary Figure 2: **FPL exponent d governed by soft particles. a** $d(\eta)$ for systems at fixed $\lambda = 1.3$. **b** $d(\lambda)$ for systems at fixed $\eta = 0.5$. Dashed curves denote fits with the fraction of the soft shells, X: $d \propto X \propto \eta$ in (A) and $d \propto X \propto (\lambda^2 - 1)/\lambda^2$ in **b**.

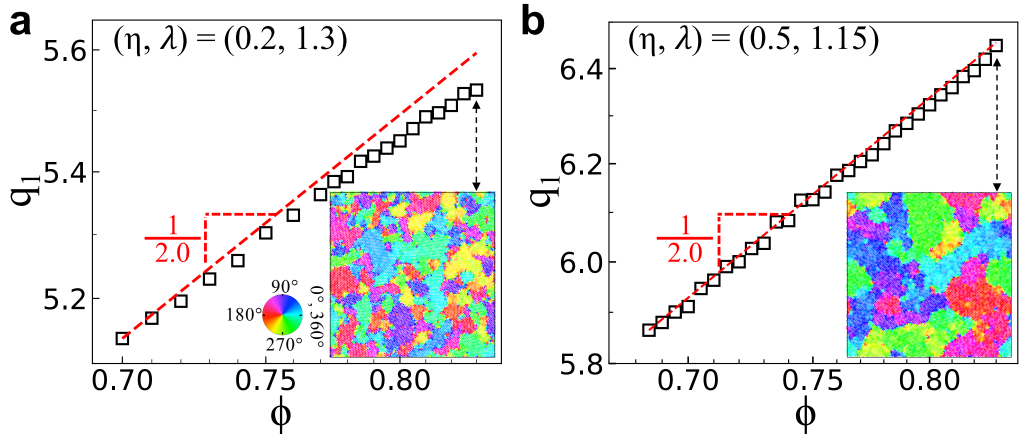

Supplementary Figure 3: **Absence of FPL (i.e. absence of $d \neq D$) in 2DSH systems that cannot be compressed into glass. a,b** Log-log plots of $q_1(\phi)$ for systems with $(\eta, \lambda) = (0.2, 1.3)$ and $(\eta, \lambda) = (0.5, 1.15)$, respectively. Insets show that the systems remain polycrystal instead of glass even at the highest pressure in our simulation. The color wheel is shown in **a**.

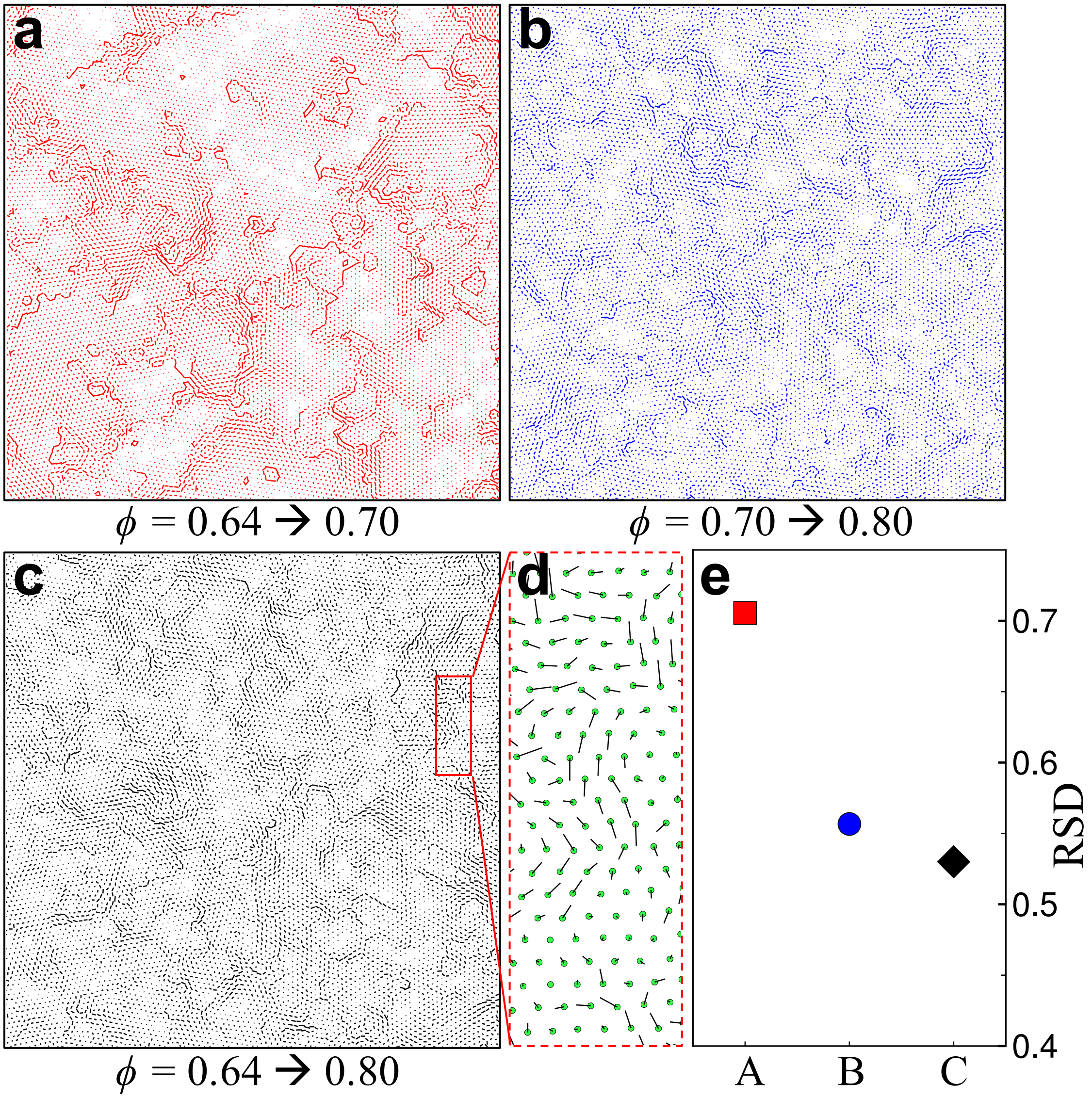

Supplementary Figure 4: **Particles displacement in 2DSH system with $(\eta, \lambda) = (0.5, 1.3)$ under compression.** **a-c** Particle displacements $\Delta\mathbf{r}$ when the system is compressed from $\phi = 0.64$ (crystal) to 0.70 (crossover state), from 0.70 (crossover state) to 0.80 (glass), and from 0.64 to 0.80, respectively. After the box sizes of the initial and final states are scaled to the same size, the position difference of jth particle gives its displacement $\Delta\mathrm{r}_j$. **d** Enlarged view of a subarea of **c**. Green circles denote the initial positions of particles. **e** The relative fluctuation of displacement, i.e. the relative standard deviation RSD $= \sqrt{\langle|\Delta\mathbf{r}|^2\rangle - \langle|\Delta\mathbf{r}|\rangle^2}/\langle|\Delta\mathbf{r}|\rangle$, for **a-c**. The large displacements in **a,b** correspond to SS and SH bonds respectively, which are anti-correlated in space. Consequently, their superposition in **c** are more uniform with less fluctuations as shown in **e**.

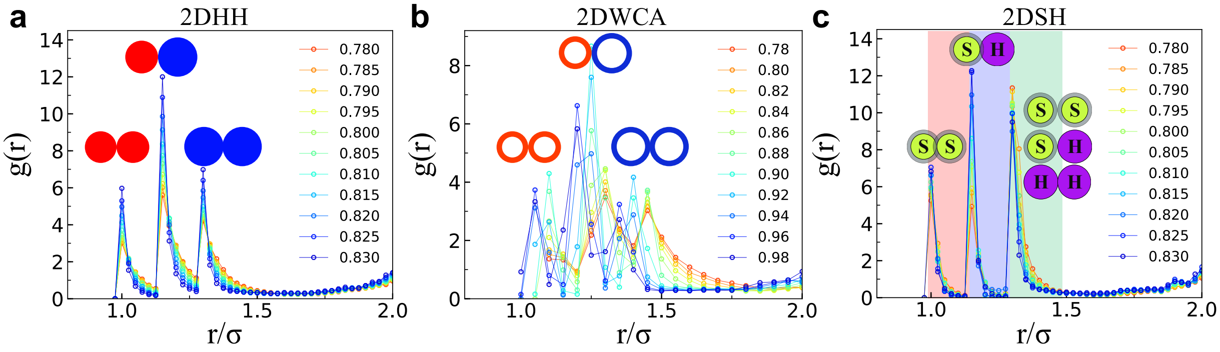

Supplementary Figure 5: **The first peak of $g(r)$ from the first-layer neighbors splits into three sub-peaks in binary systems. a-c** The radial distribution functions for 2DHH, 2DWCA, and 2DSH systems, respectively, at different ϕ. Insets are the neighboring pairs corresponding to each sub-peak. The red, blue, and green regions in **c** define the compressed SS bonds at $r < 1.14$, the compressed SH bonds at $1.14 < r < 1.28$, and uncompressed bonds at $1.28 < r < 1.5$. Once the soft shoulder is compressed, the pair distance tends to be as close as it could be, i.e. the bond is fully compressed and the inner hard core is in contact with the neighboring particle. Such configuration yields the largest free volume, thus are more favorable in entropy.

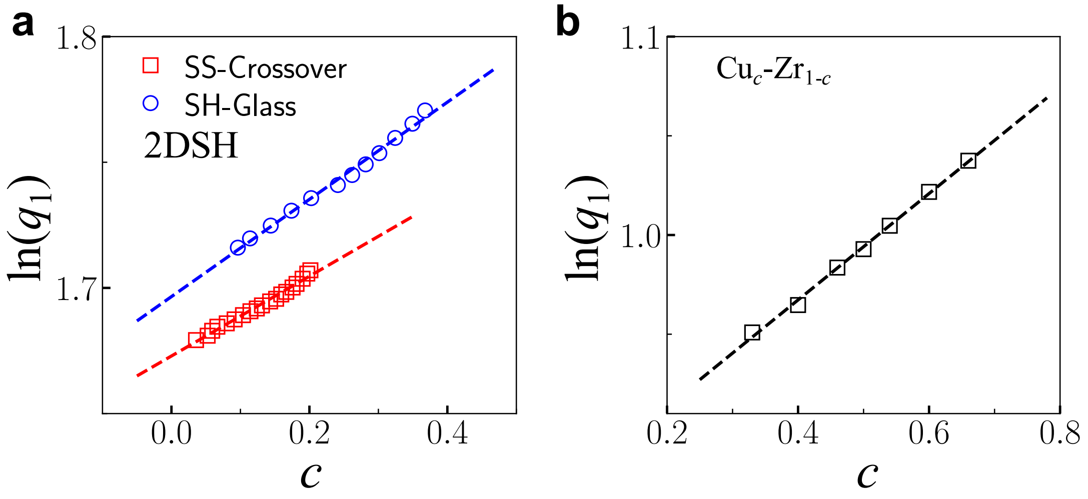

Supplementary Figure 6: **Linear relation between** $\ln(q_1)$ **and the concentration of the bonds or atoms.** **a** $\ln(q_1)$ versus the fraction of compressed SS bonds (red) and SH (blue) bonds, respectively. $c = n_{\mathrm{SS,SH}}/(3N)$. A triangular lattice with N particles has $3N$ bonds. The SS and SH bonds mainly increase at the crossover and glass regimes, respectively (Fig. 5**b**). Hence we only show them in each of these regimes. **b** $\ln(q_1)$ as function of the concentration of Cu. Data (squares) are derived from ref. [2]. Dashed lines are the linear fits.

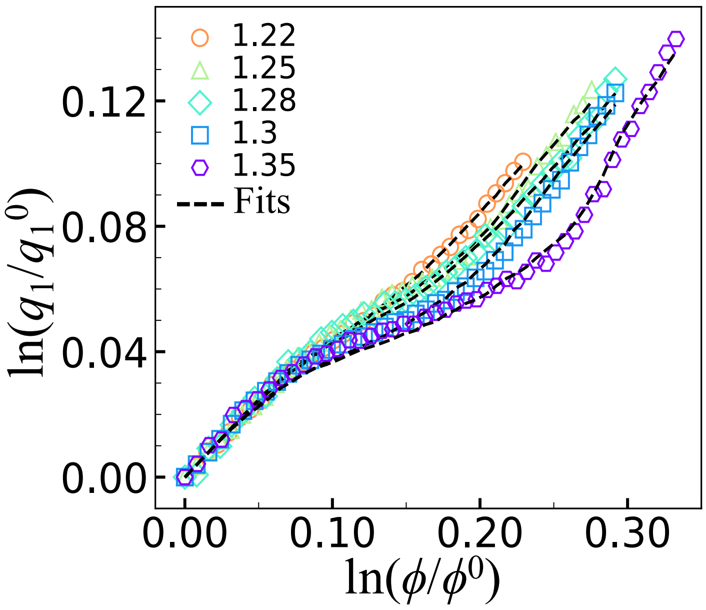

Supplementary Figure 7: $\ln(q_1/q_1^0)$ **as a function of** $\ln(\phi/\phi^0)$ **fitted with Eq. 5 for systems with** $\eta = 0.5$ **and different** λ**.**

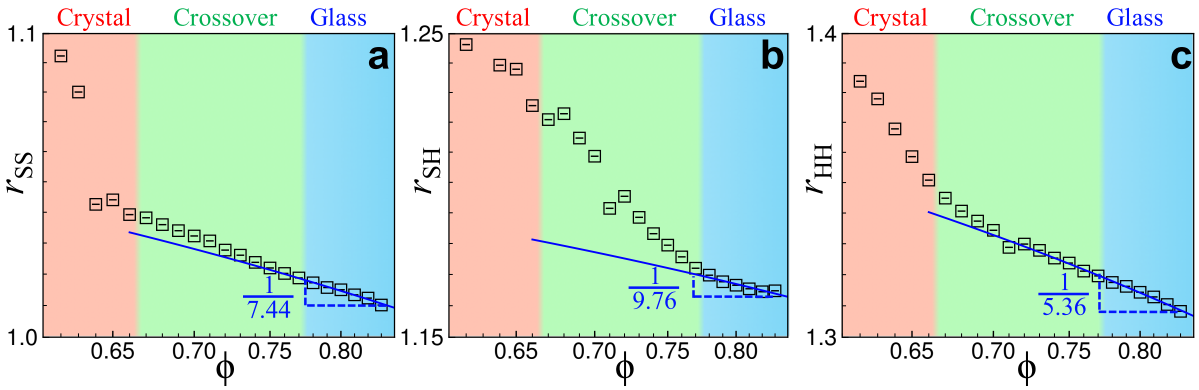

Supplementary Figure 8: **FPLs of the subpeaks in the first peak of** $g(r)$ **for 2DSH glasses with** $(\eta, \lambda) = (0.5, 1.3)$**.** $r_{\mathrm{SS,SH,HH}}$ are the mean lengths of the SS, SH and HH bonds defined in Supplementary Figure 5c. $r_{\mathrm{SS,SH,HH}} \propto \phi^{-1/d_{\mathrm{SS,SH,HH}}}$ with large exponents in the glass regime because the mean bond lengths cannot shift much, i.e. r_1 changes little. Note that if $r_{\mathrm{SS,SH,HH}}$ are defined as the subpeak positions, then the corresponding $d_{\mathrm{SS,SH,HH}}$ are even larger because each asymmetric subpeak reaches the maximum near the fully compressed bond length which is a constant.

SUPPLEMENTARY REFERENCES

[1] Zhang, H. & Han, Y. Compression-induced polycrystal-glass transition in binary crystals. *Phys. Rev. X* **8**, 041023 (2018).

[2] Calvayrac, Y., Chevalier, J. P., Harmelin, M., Quivy, A. & Bigot, J. On the stability and structure of Cu-Zr based glasses. *Philos. Mag. B* **48**, 323–332 (1983).
